# Hospitalizations for Anorexia Nervosa during the COVID-19 Pandemic in France: A Nationwide Population-Based Study

**DOI:** 10.3390/jcm11164787

**Published:** 2022-08-16

**Authors:** Jean-Christophe Chauvet-Gelinier, Adrien Roussot, Bruno Vergès, Jean-Michel Petit, Fabrice Jollant, Catherine Quantin

**Affiliations:** 1 Department of Psychiatry, Dijon University Hospital, 21000 Dijon, France; 2 INSERM, LNC-UMR 1231, University of Burgundy, 21078 Dijon, France; 3 Biostatistics and Bioinformatics (DIM), Dijon University Hospital, 21000 Dijon, France; 4 Department of Endocrinology, Diabetes and Metabolic Disorders, Dijon University Hospital, 21000 Dijon, France; 5 Department of Psychiatry and Psychotherapy, Jena University Hospital, 07743 Jena, Germany; 6 GHU Paris Psychiatrie et Neurosciences, Hôpital Sainte-Anne, CMME, 75014 Paris, France; 7 McGill Group for Suicide Studies, McGill University, Montreal, QC H3A 0G4, Canada; 8 Nîmes Academic Hospital (CHU), 30900 Nîmes, France; 9 Moods Team, INSERM, UMR-1178, CESP, 94276 Le Kremlin-Bicêtre, France; 10 INSERM, CIC 1432, Clinical Investigation Center, Clinical Epidemiology/Clinical Trials Unit, Dijon University Hospital, 21000 Dijon, France; 11 INSERM, CESP, UVSQ, Université Paris-Saclay, 94807 Villejuif, France

**Keywords:** anorexia nervosa, hospital data, mental health, self-harm, eating disorders

## Abstract

The COVID-19 pandemic has had a detrimental impact on mental health, including on food-related behaviors. However, little is known about the effect of the pandemic on anorexia nervosa (AN). We sought to assess an association between the COVID-19 pandemic and a potential increase in hospitalizations for AN in France. We compared the number of hospitalizations with a diagnosis of AN during the 21-month period following the onset of the pandemic with the 21-month period before the pandemic using Poisson regression models. We identified a significant increase in hospitalizations for girls aged 10 to 19 years (+45.9%, RR = 1.46[1.43–1.49]; *p* < 0.0001), and for young women aged 20 to 29 (+7.0%; RR = 1.07[1.04–1.11]; *p* < 0.0001). Regarding markers of severity, there was an increase in hospitalizations for AN associated with a self-harm diagnosis between the two periods. Multivariate analysis revealed that the risk of being admitted for self-harm with AN increased significantly during the pandemic period among patients aged 20–29 years (aOR = 1.39[1.06–1.81]; *p* < 0.05 vs. aOR = 1.15[0.87–1.53]; NS), whereas it remained high in patients aged 10 to 19 years (aOR = 2.40[1.89–3.05]; *p* < 0.0001 vs. aOR = 3.12[2.48–3.98]; *p* < 0.0001). Furthermore, our results suggest that the pandemic may have had a particular effect on the mental health of young women with AN, with both a sharp increase in hospitalizations and a high risk of self-harming behaviors.

## 1. Introduction

Mounting evidence suggests that the 2019 coronavirus (COVID-19) pandemic had an adverse impact on the mental health of populations around the world [1,2]. In early 2020, a few months after the first appearance of the SARS-CoV-2 infection in the Chinese city of Wuhan, most governments decided to impose national lockdowns and social restrictions in order to stop the spread of the COVID-19 epidemic. In France, to tackle the growing COVID-19 pandemic, a first national lockdown was implemented from 17 March to 11 May 2020, with restriction measures gradually raised in summer 2020. Subsequently, in fall of 2020, a four-week partial lockdown (with schools remaining open) was enforced from 30 October until 15 December to combat the second wave of COVID-19. It is worth mentioning that various restrictions remained in place until June 2021, such as nighttime curfews and restricted mobility, as well as recurrent closures of classrooms when there were COVID-positive cases. Ultimately, this period from March 2020 to June 2021 corresponded to nearly 12 months of disrupted lifestyles, social restrictions and uncertainty about the future, with a likely negative impact on mental health.

Although the French authorities actively supported the economy and therefore indirectly limited the risk of an economic recession that may have had additional negative psychological consequences, the national containment measures, which included social distancing, as well as the closure of schools, universities, and “non-essential” workplaces and services, led to social isolation and loneliness for many people. Numerous reports have described the increase in psychological disturbances (i.e., anxiety, depression and self-harming behaviors) that emerged during the COVID-19 crisis [3,4,5,6,7,8,9,10].

Some research has suggested that the pandemic may have had an impact on eating disorders [11]. Anorexia nervosa (AN) is a severe form of eating disorder that is often associated with anxiety, mood disorders and suicide attempts [12,13]. Moreover, AN is one of the most life-threatening mental disorders, especially for young women [12]. In contrast to other psychiatric illnesses, the years lived with disability have increased in AN over time [12]. In addition, the AN-related death rate remains very high. Recent investigations have documented a five-fold standardized mortality ratio (SMR) after inpatient treatment for AN in comparison to age-matched and gender-matched individuals in the general population [14,15]. However, there are conflicting data with respect to the impact of the pandemic on eating disorders. Several reports showed clinical improvements and reductions in hospitalizations after lockdown for patients with AN [16,17], whereas other research suggests deleterious effects on young patients with AN [18,19,20]. It thus seemed relevant to further investigate the influence of the COVID-19 pandemic on people with AN [21]. One could speculate that the negative impacts of confinement [22] which are generally described in mental disorders might have been aggravated for people with AN [23]. In general, the COVID-19 pandemic has been associated with changes in dietary habits and health behaviors, with an increased risk of weight gain due to unbalanced diets and decreased physical activity [24]. Whereas these unhealthy changes were reported in the general population, they may have elicited particularly deleterious responses in the population with AN, which is highly sensitive to issues of dietary control and energy expenditure [11].

In order to explore the potential negative impact of the COVID-19 pandemic on patients with AN, we examined trends in the number of hospitalizations of patients with AN among French people before and during the COVID-19 pandemic. The present study was based on analysis of exhaustive national data including 21,600 French people hospitalized for AN during the period of 2018–2021. Furthermore, as highlighted by recent work showing that major complications of AN manifest most often as serious medical problems and self-harming behaviors, leading to hospitalization [25,26], we also examined associated clinical markers of severity, i.e., intensive care unit admissions, self-harm and death during hospitalization. We hypothesized that the first wave of COVID-19 might be associated with an initial reduction in hospitalizations, followed by an increase during the subsequent waves of the pandemic, as illustrated by recent research on self-harm hospitalization [27] and related to delayed psychological distress in young people. We also hypothesized that there would be an increase in indirect severity markers during the COVID-19 pandemic.

## 2. Materials and Methods

### 2.1. Study Design

We conducted an observational, retrospective, population-based study comparing the number of hospitalizations for AN and their characteristics in France over two 21-month periods: before (1 June 2018–29 February 2020) and during (1 March 2020–30 November 2021) the COVID-19 pandemic. The number of hospitalizations between the two periods were compared, and multivariable models were applied in order to understand factors associated with several adverse outcomes, such as hospitalization in an intensive care unit (ICU) or diagnosis of self-harm during a hospitalization for AN.

### 2.2. Source of Data and Selection Criteria

All data were extracted from the French national hospital discharge database (Programme de Médicalisation des Systèmes d’Information, PMSI), which compiles all information on hospital stays in France.

We identified all hospital discharge abstracts registered between 1 June 2018, and 30 November 2021, with the following diagnosis codes from the International Classification of Diseases-Tenth Revision (ICD-10): F50.0 (anorexia nervosa) or F50.1 (atypical anorexia nervosa). We included hospital stays registered in both public and private hospitals in France (including overseas territories) for patients aged 10 years or older admitted to medicine/surgery/obstetrics wards. Data on inpatient stays were exclusively from general inpatient units, including emergency and medical services, but not including psychiatric hospitalizations.

### 2.3. Variables

The main outcome was the number of hospital stays with a diagnosis of AN.

Hospital stays were further grouped into two cohorts: hospitalizations for AN before the pandemic (from 1 June 2018 to 29 February 2020), and hospitalizations for AN after the beginning of the pandemic (from 1 March 2020 to 30 November 2021).

We then compared several variables of interest between the two time periods: age at hospitalization grouped into classes (10–19 years old, 20–29, 30–39, 40–49, 50–59, and 60 and older), sex, number of patients, number of stays per patient and mean duration of stay in days. Several proxies of severity [25,26] were also extracted, including hospitalizations for AN with a diagnosis of intentional self-harm (ICD-10 codes X60–X84), hospitalizations for AN in an ICU and death at discharge.

**Figure 1:**
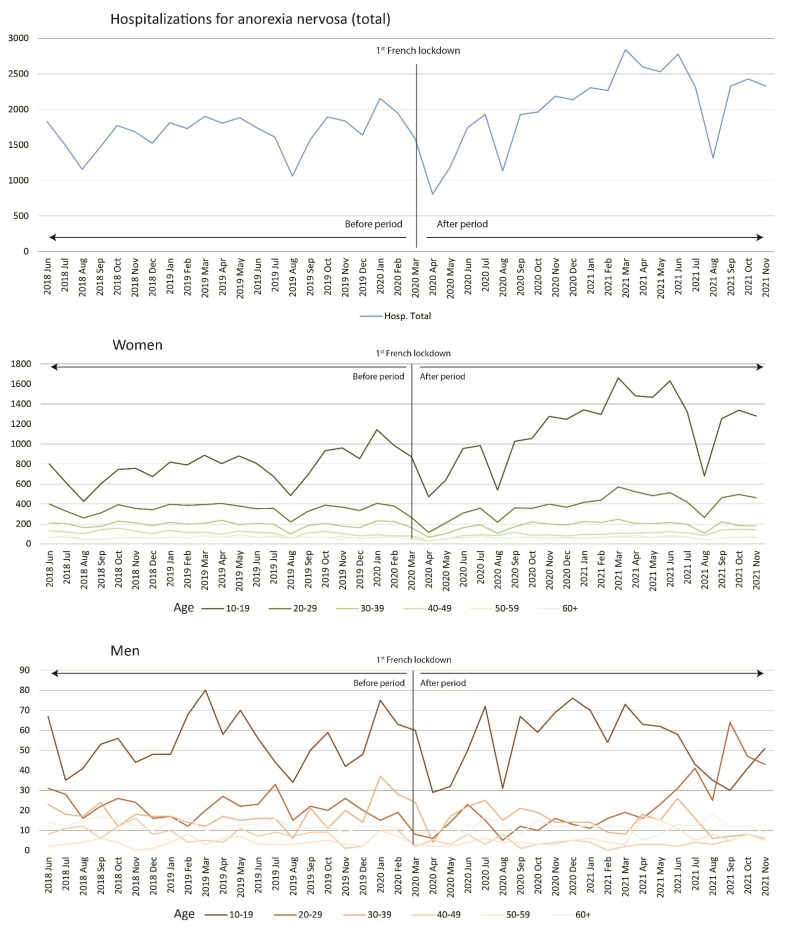
Number of monthly hospitalizations for patients with anorexia nervosa from June 2018 to November 2021: overall (**top**), in women by age group (**middle**) and in men by age group (**bottom**).

**Figure 2:**
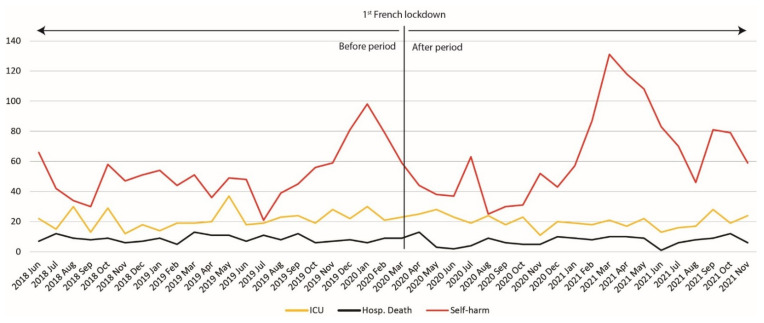
Monthly number of hospitalizations for patients with anorexia nervosa with an associated code for self-harm, admission to an intensive care unit or with hospital death from June 2018 to November 2021.

Table 1 presents the number of stays for AN for each of the variables studied during the period; Figure 1 and Figure 2 show the number of hospitalizations for AN by sex, age group and severity marker during the studied periods.

### 2.4. Statistical Analysis

Differences in the number of hospital stays for AN between the two periods were estimated using relative risk (RR) obtained by modified Poisson regression models and presented with 95% confidence intervals (CIs). A modified Poisson regression model was described by Zou [28], who suggested using a modified Poisson approach to estimate relative risk with robust error variances. Mean age and length of hospital stays were compared using Student’s *t* tests. The difference in hospital mortality between the two periods was assessed using a Cox proportional-hazards regression model considering the length of stay for patients who died in hospital. We also applied four logistic models (binary logit models) to investigate factors associated with severity markers: hospitalization for AN in ICU and hospitalization for AN associated with an intentional self-harm code. The dependent variables were sex, age and the other severity markers when applicable: self-harm hospitalization, hospitalization in ICU and death at discharge. The results of logistic models are expressed as adjusted odds ratios (ORs) and presented with their 95% confidence intervals (CIs).

All analyses were performed using SAS (SAS Institute Inc, Version 9.4, Cary, NC, USA). The Poisson regression models were estimated using the GENMOD procedure with a “repeated” statement. Logistic models were performed using the LOGISTIC procedure.

## 3. Results

The general characteristics of the study population are presented in Table 1. From June 2018 to November 2021, there were 78,179 hospitalizations for AN in France, corresponding to 21600 patients. As expected with AN, the vast majority of patients were women (93.8%).

### 3.1. During vs. before the COVID-19 Pandemic

In overall terms, there was a 20% increase in hospitalizations for AN during the pandemic period compared with the preceding period (Table 1). The number of patients admitted to hospital with AN also increased by 9%. We found no difference in the rate of hospitalization for men with AN during the pandemic, but for women, there was a significant increase of 21.98%.

However, this overall increase in admissions during the pandemic was not observed in women of all age categories. There was a considerable increase of 45.9% among girls with AN aged 10–19 years (RR = 1.46[1.43–1.49]; *p* < 0.0001) and a substantial increase of 7.0% in young women aged 20–29 (RR = 1.07[1.04–1.11]; *p* < 0.0001). In contrast, there was a slight decrease in admissions of women aged 30–49 and no significant change in women over 50.

Regarding markers of severity, there was a 23.3% increase in hospitalizations for self-harm among patients with AN. In contrast, there was a 5.3% reduction in the number of admissions to ICUs and a 14.9% decrease in hospital deaths in patients with AN during the pandemic period.

### 3.2. Monthly Changes

When we assessed the monthly variations in hospitalizations for AN from June 2018 through November 2021, we noticed a decline in spring 2020 during the initial lockdown (March–April 2020) and a subsequent increase in admissions until the end of the study period (with the exception of the summer periods in 2020 and 2021) (Figure 1).

In terms of variations in hospitalizations for AN by age and gender, there was a similar decrease in hospitalizations among women aged 10–19 years and, to a lesser extent, among those aged 20–39 years at the beginning of the lockdown period, followed by an almost constant increase in hospitalizations until the end of the study period (with the exception of the summer periods in 2020 and 2021).

Furthermore, when considering the admissions of patients with AN in terms of markers of severity (Figure 2) in the two periods, we observed a seasonal pattern of self-harm hospitalizations: a decrease during the first half of 2020 followed by an increase from September 2020 to March 2021, another decrease during summer 2021 and another increase in September-October 2021.

The evolution over time was more variable for intensive care hospitalizations and in-hospital deaths.

### 3.3. Multivariate Analysis: Risks of ICU Admissions and Hospitalization for AN with a Self-Harm Diagnosis

#### 3.3.1. Factors Associated with ICU Admission of Patients with AN

Regarding the risk of admission to an ICU for patients with AN, there was a decrease in risk for all age groups during the pandemic period, irrespective of sex. Regarding other adverse outcomes associated with admission to ICUs, the risk of self-harm and in-hospital death decreased significantly (Table 2).

#### 3.3.2. Factors Associated with Admissions of Patients with AN for Self-Harm

The risk of hospitalization for self-harm decreased after the beginning of the pandemic in patients with AN aged 10 to 19 years (aOR = 2.40[1.89–3.05]; *p* < 0.0001 vs. aOR = 3.12[2.48–3.98]; *p* < 0.0001), but the risk was still significant and high in comparison to other age groups. The risk of associated self-harm hospitalization became significant in people aged 20 to 29 years during the pandemic period (aOR = 1.39[1.06–1.81]; *p* < 0.05 vs. aOR = 1.15[0.87–1.53]; NS). However, the risk of hospitalization for self-harm remained low and stable in patients with AN aged 40 to 49 years and decreased significantly in patients over the age of 50 (Table 2).

Regarding gender, the excess risk of hospitalization for self-harm was significant among women with AN and increased by 1.1 during the pandemic compared to men (Table 2).

The association between self-harm and ICU admissions decreased slightly during the pandemic period. Lastly, there was no significant change in hospital deaths associated with self-harm (Table 2).

## 4. Discussion

The present study is the first to examine a potential relationship between the COVID-19 pandemic and hospitalizations for anorexia nervosa, providing a comparative hospital survey between the pre-pandemic period (2018–early 2020) and the pandemic period (2020–2021) based on the French National Health Data System. As hypothesized, at the beginning of the first COVID-19 wave and the first lockdown in March 2020, there was an initial moment of panic during which non-COVID-19 hospital activity collapsed, including hospitalizations for self-harm associated with anorexia nervosa [3,6,27,29,30]. This considerable decrease was not followed by a sustained reduction in inpatient admissions for anorexia nervosa. Instead, after an ephemeral drop toward baseline, we noticed a substantial increase in hospitalizations for AN, with a significant increase in adolescent girls aged 10 to 19 years and young women aged 20 to 29. This is congruent with studies showing that adolescent mental health has been considerably affected by the pandemic [18,20,30], with an impact that was first internalized (e.g., anxiety, depression and eating disorders) and then externalized (e.g., hospitalizations for AN) [31]. It has been suggested that during the period of isolation, people with AN may have been forced to change their eating routine, to isolate themselves socially or, conversely, to be confined with their families [11,23]. Other authors have argued that the alternating of periods of lockdown with gym closures and the associated psychosocial disruption of patient routines altered their ability to exercise, thereby enhancing their concern about weight gain in a constrained household environment. The pandemic represented a sudden and profound disruption of lifestyle. It was certainly a potential stressor, leading to aggravation of AN or even onset of the illness in some vulnerable individuals [32]. In addition, lockdowns, school closures, curfews and telecommuting may have resulted in closer contact between parents and children during the pandemic, allowing parents to pay more attention to their children’s health. Solmi et al., reiterated the crucial role that parents play in the early management of eating disorders, so it is possible that parents were instrumental in prompting hospital admission for young patients in this particular context [33]. However, a reduction in all hospital admissions for all diagnoses was observed at the beginning of the pandemic for both adults and children. This raises the question of whether it was only the most severe cases that came to the hospital. In our study, in line with the results reported by Goldberg et al. [17], this increase in hospitalizations for AN did not seem to be associated with increased medical severity (i.e., ICU admission and lethality). Moreover, bearing in mind the overall decreases in psychological health, we found that admissions for self-harm in young patients with AN increased in the post-lockdown period, with a peak in the winter of 2020–2021. This seems to be in line with the results of several research studies pointing out the deterioration of mental health in adolescents and young adults after the first wave of the pandemic, especially in the aftermath of the second wave in late 2020 [21,31]. On the contrary, we may wonder whether this significant increase in hospitalizations of adolescent girls with AN corresponds to the emergence of new patients with this eating disorder. The pandemic might have been a factor likely to reveal a vulnerability to eating disorders. The average age of onset of AN symptoms is between 14 and 19 years [34], and the spectacular increase in the number of hospitalizations among girls under the age of 19 years in this study might suggest an elevated incidence of these eating disorders during the pandemic [33]. Our multivariable analysis highlights such information and shows an increased risk of hospitalization for AN and self-harm during the pandemic compared to the 2018–2020 period among both adolescents under 19 years of age and in young adults aged 20–29. This result appears to be consistent with previous analyses highlighting the psychological distress associated with the pandemic [35]. Several studies demonstrated a clear reduction in hospital discharges due to severe mental health conditions (i.e., self-harm) at the beginning of COVID-19 pandemic, followed by increased suicidal behaviors and hospitalizations in the summer of 2020 [27,29]. Moreover, this deterioration in mental health associated with AN is consistent with the pathophysiology commonly evoked in the field of anorexia nervosa, such as a vulnerability to acute stressful experiences (e.g., the COVID-19 pandemic) [32]. Likewise, anorexia nervosa is inherently linked to a very high risk of suicidal behavior outside of any pandemic [36]. The results of our study therefore call for special attention to this eating disorder, in which self-harming behaviors increased significantly during the pandemic. This result is consistent with other recent reports showing a marked increase in self-harm among young people younger than 24 years old [30]. The present study makes an additional contribution by clarifying the pattern of mental health problems in the successive phases of the COVID-19 pandemic in France, i.e., a sharp deterioration in the mental health of girls and young women in terms of anorexia nervosa in the second half of 2020 and throughout 2021.

Our study has some limitations, primarily due to its hospital database analysis design, which is based on ICD-10 diagnosis codes. The clinical symptoms and specific patient characteristics were not available in the PMSI database, which limits the scope of the analyses and interpretation of the results. In addition, we cannot dismiss the possibility that patients with AN were managed by ambulatory physicians, thereby reducing hospital care. For instance, it has been shown that the prescriptions of psychotropic drugs, such as antidepressants, anxiolytics and hypnotics, increased dramatically during the pandemic nationwide [7,37], suggesting an increase in outpatient versus inpatient care. In addition, we must point out that the study was carried out exclusively on inpatients with AN admitted to general hospital wards, at least initially, which could potentially lead to an underestimation of the number of patients with AN hospitalized exclusively for psychiatric stabilization (i.e., in psychiatric units, which are separate from the other hospital wards). Nevertheless, AN is an integrative somatopsychic pathology in which acute progression is often associated with a deterioration of physical health and/or suicidal behavior, which we were unable to exhaustively measure through our analysis of national medical databases. Finally, any attempt to generalize the conclusions of this French national survey should be made with caution, considering that the psychosocial impact of the pandemic can vary depending on the culture, healthcare system and policy strategies implemented in each country [2].

## 5. Conclusions

Whereas many studies have suggested that the mental health repercussions of COVID-19 could reach or even surpass the epidemic concern in numerous countries, this study sheds light on the probable psychological consequences of the first two waves of COVID-19 in a Western country. This exhaustive nationwide exploration of 78,179 hospitalizations for anorexia nervosa from June 2018 to November 2021 identified a sizable increase in admissions among adolescents and young women in 2020 and 2021. We also provide new information on the severe psychological impact of the COVID-19 pandemic, particularly regarding concomitant self-harm. Our report underlines the importance of continued research and should encourage French health authorities to take a closer look at the mental health effects of the COVID-19 pandemic. Further analyses are needed to examine the influence of the pandemic on psychiatric outcomes and to clarify the multifactorial determinants that link the pandemic to psychological distress in populations who have or who are at risk of developing an eating disorder.

## Figures and Tables

**Table 1:** Number of hospitalizations according to age and gender, as well as hospitalization characteristics and comparisons between before and during the COVID-19 pandemic periods.

	Pre-COVID-19		During COVID-19		Total		Relative Risk (95% CI) for the pre- vs. during-COVID-19 Periods		
	June 2018–Feburary 2020		March 2020–November 2021						
	N	%	N	%	N	%			
**Hospitalizations**	35,536		42,643		78,179		1.20 (1.18–1.22) ***		£
**Patients**	10,330		11,270		21,600		1.09 (1.06–1.12) ***		
**Gender and age**									
Female	33,045	*93.0*	40,307	*94.5*	73,352		1.22 (1.20–1.24) ***		£
Mean age	25.6 (±14.6)		23.4 (±13.2)				*t*-Test = −21.9; *p* < 0.0001		$
10–19	16,338	*46.0*	23,833	*55.9*	40,171	*51.4*	1.46 (1.43–1.49) ***		£
20–29	7489	*21.1*	8016	*18.8*	15,505	*19.8*	1·07 (1.04–1.11) ***		
30–39	4125	*11.6*	3786	*8.9*	7911	*10.1*	0.92 (0.88–0.96) ***		
40–49	2367	*6.7*	2063	*4.8*	4430	*5.7*	0.87 (0.82–0.93) ***		
50–59	1380	*3.9*	1344	*3.2*	2724	*3.5*	0.97 (0.90.1.05)		
60+	1346	*3.8*	1265	*3.0*	2611	*3.3*	0.94 (0.87–1.02)		
Male	2491		2336		4827		0.94 (0.89–0.99) *		£
Mean age	29.3 (±19.0)		28.8 (±18.6)				*t*-Test = -0.9; *p* = 0.302		$
10–19	1139	*2.6*	1125	*3.2*	2264	*2.9*	0.99 (0.91–1.07)		£
20–29	454	*1.1*	458	*1.3*	912	*1.2*	1.01 (0.89–1.15)		
30–39	373	*0.7*	308	*1.1*	681	*0.9*	0.83 (0.71–0.96) *		
40–49	171	*0.2*	82	*0.5*	253	*0.3*	0.48 (0.37–0.62) ***		
50–59	88	*0.3*	123	*0.3*	211	*0.3*	1.40 (1.06–1.84) *		
60+	266	*0.6*	240	*0.8*	506	*0.6*	0.90 (0.76–1.07)		
**Hosp. Characteristics**									
Stays per patient	3.44 (±8.4)		3.78 (±10.5)		-		*t*-Test = 2.63; *p* = 0.008		$
Mean length of stay (days)	5.32 (±16.3)		5.37 (±17.4)		-		*t*-Test = 0.49; *p* = 0.627		
Self-harm	1088	*3.1*	1341	*3.1*	2429	*3.1*	1.23 (1.14–1.34) ***		£
Hosp. In ICU	452	*1.3*	428	*1.0*	880	*1.1*	0.95 (0.83–1.08)		
Hosp. Death	181	*0.5*	154	*0.4*	335	*0.4*	HR = 0.73 (0.59–0.91); *p* = 0.004		µ

Footnotes: CI: confidence interval; ICU: intensive care unit; * *p* < 0.05; *** ≤0.0001. £: Poisson regression model; $: T-test; µ: Cox model.

**Table 2:** Risk of admission to an intensive care unit or self-harm diagnosis associated with hospitalization for AN (adjusted odds ratios before and after beginning of the COVID-19 pandemic).

	Hospitalization in Intensive Care Unit (N = 880)				Self-Harm Diagnosis during Hospitalization for AN (N = 2429)			
	BEFORE COVID-19 (N = 452)		DURING COVID-19 (N = 428)		BEFORE COVID-19 (N = 1088)		DURING COVID-19 (N = 1341)	
	N	aOR	N	aOR	N	aOR	N	aOR
**Age class**								
10–19	55	0.16 (0.11–0.23) ***	73	0.11 (0.08–0.15) ***	771	3.12 (2.48–3.98) ***	981	2.40 (1.89–3.05) ***
20–29	108	0.89 (0.65–1.22)	99	0.50 (0.37–0.67) ***	150	1.15 (0.87–1.53)	212	1.39 (1.06–1.81) *
30–39	Ref				Ref			
40–49	58	1.53 (1.07–2.19) *	56	1.22 (0.86–1.72)	39	0.85 (0.57–1.27)	38	0.84 (0.56–1.25)
50–59	71	2.86 (2.02–4.06) ***	52	1.61 (1.13–2.30) **	35	1.11 (0.73–1.69)	16	0.49 (0.28–0.85) *
60+	91	2.97 (2.10–4.19) ***	58	1.45 (1.01–2.09) *	19	0.53 (0.31–0.89) *	14	0.45 (0.25–0.81) **
**Gender**								
Female	422	1.32 (0.89–1.96)	392	0.81 (0.57–1.17)	1058	2.58 (1.79–3.73) ***	1321	3.71 (2.38–5.80) ***
**Other adverse outcome**								
Hosp. death	54	13.94 (9.63–20.18) ***	28	8.42 (5.30–13.38) ***	7	0.62 (0.27–1.43)	1	0.20 (0.03–1.46)
Self-harm	85	14.70 (11.22–19.25) ***	85	14.26 (10.96–18.55) ***				
ICU					85	15.05 (11.44–19.81) ***	85	14.68 (11.27–19.13) ***

Footnotes: I: Intensive Care Unit; N: number of hospital stays; aOR: adjusted odds ratio; * *p* < 0.05; ** *p* ≤ 0.01; *** *p* ≤ 0.0001.

## Data Availability

The PMSI database was made available by the French National Agency for the Management of Hospitalization Data (Agence technique de l’information sur l’hospitalisation, ATIH). The use of these data by our department was approved by CNIL. We are not permitted to share these data. PMSI data are available from ATIH to researchers who meet the criteria for access (requests for access are evaluated by the CNIL).
